# A new R-based statistical software package for fitting multivariate generalized linear mixed models to large and complex datasets

**DOI:** 10.23889/ijpds.v1i1.355

**Published:** 2017-04-19

**Authors:** Damon Berridge, Robert Crouchley, Daniel Grose

**Affiliations:** 1 Swansea University Medical School; 2 Lancaster University Management School

## Objective

To demonstrate the efficiency and efficacy of the new freeware MGLMM in the statistical modelling of large and complex datasets.

## Approach

The new R-based software MGLMM (shortly to become available on the CRAN website https://cran.r-project.org/ ) can be used to fit a range of different multivariate generalized linear mixed models, thereby permitting a better representation of the multivariate nature of many complex socio-economic processes which may involve a range of different response types. The software MGLMM will also set records for the time taken to fit these complex models to large sets of data. The efficiency and efficacy of this new software will be demonstrated by way of a number of applications.

One such example will come from the STAR Project in which pupils were followed from kindergarten in 1985 (aged 5) to 1989 (third grade, aged 8). The pupils were assessed on their ability in both mathematics and English on an annual basis between 1985 and 1989. At the end of the kindergarten year, some of the students were reallocated to different class sizes in order ‘to achieve sexual and racial balance and to separate incompatible children’. In the fourth grade, the pupils returned to regular classes and the experiment ended.

We can regard the two score variables `Maths' and `English' as constituting repeated, correlated, bivariate continuous data. We will use the new package MGLMM to apply a series of bivariate normal mixed models with correlated random effects. These models will allow us to distinguish the role of prior attainment in mathematics and English on current attainment in mathematics and English in an experimental setting, for pupils in small and regular class sizes. The models will permit an examination of the effect of class size on this bivariate response, whilst controlling for a set of individual-level confounding factors such as gender and age.

## Results

We will present the results of these analyses, thereby demonstrating the efficacy of MGLMM. We will also compare the performance of the new software with existing packages such as Stata. 

## Conclusions

We will have demonstrated the gain in efficiency through the use of MGLMM, compared to other standard software packages, in the statistical modelling of large and complex datasets.

